# Supplementary material for: De Novo Assembly of the Trypanosoma congolense Genome Reveals an Organization Influenced by Antigenic Variation but Distinct from Trypanosoma brucei
Source: Genome Biol Evol. 2026 Aug 25;18(9):evag213. doi: 10.1093/gbe/evag213 (PMC13547854; doi:10.1093/gbe/evag213)
Supplement: Supplementary file 1 [file evag213_supplementary_data.pdf]

Supplementary figure and table legends

Figure S1. Confirmation of TcoRAD51 and TcoBRCA2 null mutants. A. Diagram illustrating the insertion of pJM-RMC-09 or -19 into the native *TcoRAD51* locus; primer binding sites are indicated by coloured arrowheads, and the gel image shows PCR using these primers to test for loss of the TcoRAD51 ORF in wild type cells (WT), single allele knockout cells (sKO), and double allele knockout cells (dKO). **B.** As in A, but for *TcoBRCA2*. **C.** RT-qPCR of *TcoRAD51* and *TcoBRCA2*, showing expression of each gene relative to that in WT for both TcoRAD51$^{-/-}$ H2/B2 and TcoBRCA2$^{-/-}$ Fx 2 (double allele KO cells). The mean of 4 experiments (each of 3 replicates) is shown ±SD.

Figure S2. Summary of evaluated genome assemblies. During the genome assembly process, several assemblies were produced using a range of data combinations, assembly tool settings, and two assemblers. Numbers 1-15 refer to the combinations used. **A.** The upset plot highlights the various combinations of data and settings used in assembly attempts for *T. congolense*, showing the two assemblers – hifiasm and verkko – in separate panels. P0 refers to refers to long read ONT data collected from wild type cells at passage 0 (prior to prolonged *in vitro* growth of wild-type clones 1 and 2). **B.** Breakdown of how many contigs in each size category were retrieved in each assembly attempt, and whether the contigs contained both, one, or no telomeric ends (TTAGGG), based on the output of seqtk telo. The y axis represents the different assemblies, 1-15. **C.** BUSCO results. **D.** QV scores for the 15 assemblies.

Figure S3. Chromosome pairing of the largest 13 *T. congolense* chromosomes. The figure highlights the best within-genome matches for the largest 28 contigs (excluding self-matches), allowing the identification of homologous chromosome pairs (data generated using nucmer of mummer suite of tools, and identical top matches in 1 kbp bins have been merged). The four copies of chromosome 1 (tco01a-tco01d) have an unusual appearance due to different regions of the contigs having different best matches among the other three copies. Chromosome 4 (Tco04a and tco04b) shows more limited homology compared to other chromosomes.

Figure S4. Gene organisation in the 13 largest chromosomes of *T. congolense*. All assembled chromosomes of *T. congolense* IL3000 above 850 kbp in length are shown, comparing two haplotypes for chromosomes 2-13 and four haplotypes for chromosome 1. Genes and pseudogenes predicted using Companion(Haese-Hill, et al. 2024) are shown in grey, with their coding strand indicated relative to a central line; *VSG* family genes are highlighted (yellow for Pfam PF00913, green for Pfam PF13206). An unresolved sequence gap is highlighted by an asterisk.

Figure S5. Chromosome copy analysis for chromosomes 2-3 and 5-13. Panels show the estimated chromosome copy number for chromosomes not shown in Figure 2. Data for wild-type, *BRCA2*-null and *RAD51*-null cells is shown; P0 and P22 refer to passages during prolonged *in vitro* growth.

Figure S6. *VSG* family genes in the largest 13 *T. congolense* chromosomes. A. Distribution of VSG family Pfam-containing genes and pseudogenes in the larger chromosomes (PF00913, VSG A; PF13206, VSG B Pfam domains). **B.** Distribution of phylotypes among the putative *VSG* genes (based on VAPPER Pfam domains(Silva Pereira, et al. 2019)). The 'undetermined' category contains sequences that either do not have a significant phylotype motif hit, or more than one motif is detected above the significance threshold. The number of *VSG*s for each chromosome contig is shown above the plot. **C.** Gene content of the two haplotyps of chromosome 4 (tco04a and tco04b) based on Pfam analysis; numbers in brackets indicate the number of genes and pseudogenes, as determined by pfam_scan (note – pfam_scan uses different thresholds for detecting sequence motifs than hmmer, which was used for detecting Pfam domains for panels A and B).

Figure S7. Gene expression of the largest *T. congolense* chromosomes. Bulk RNAseq data is shown mapped to all copies of the largest *T. congolense* contigs (>850 kbp), with VSG family Pfam-containing genes and AT-rich tandem repeat regions highlighted. RNAseq was obtained from wild-type cells and analysed using Salmon (output in TPM).

Figure S8. Contact decay between chromosome homologues. Contact decay for haplotype A (hapA) and haplotype B (hapB) chromosome contigs at different resolutions (25, 50 and 100 kbp, as plotted by hicPlotDistVsCounts of HiCExplorer suite of tools).

Figure S9. Correlation matrices for chromosomes 1-3, 6-9, 11-12. Correlation matrices at 25 kbp resolution for haplotype A chromosome contigs not presented in Figure 4B. PC1-4 plotting range is -0.1 to +0.1, insulation score plotting range is -2 to +2. PC denotes principal component, IS – insulation score.

Figure S10. Corrected interaction matrices for the 13 largest chromosomes of *T. congolense*. Corrected matrices are shown at 25 kbp resolution for all haplotype A chromosome contigs (tco01a-tco13a), with predicted genes highlighted in black and grey (gene strand), putative *VSG*s shown in green, and AT-rich tandem repeats (putative centromeric repeats) shown in light blue. Data filtered out due to multimapping appears in white.

Table S1. QUAST metrics for *T. congolense* IL3000 genome assemblies. Various genome assembly metrics, as reported by QUAST, are shown for the two previously published *T. congolense* IL3000 genomes ('IL3000' and 'IL3000_2019'), as well as the genome assembly presented here ('2026'). 'IL3000' corresponds to TriTrypDB build version 68 assembly 'TriTrypDB-68_TcongolenseIL3000', and 'IL3000_2019' corresponds to 'TriTrypDB-68_TcongolenseIL3000_2019'.

Table S2. Genome completeness metrics and contig breakdown by type. Assembly completeness metrics were performed on both assemblies that were combined to produce the final assembly, as well as the final combined assembly. Mapped read percentage was retrieved using samtools flagstat.

Table S3. Primers Used

Primer Name	Gene/Purpose	Sequence	Restriction site
JM010	Blasticidin R	TCTAGAGCCCTCCCACACATAACC	
JM014	HSV screen F	CTGGGCCTTGGACGTCTTGG	
JM015	KO plasmid seq 5'	CGACCGAGCGCAGCGAGTCA	
JM016	RAD51 5UTR F	gcggccgcGATGTGCCCATTTGTGTCAC	*Not*I
JM017	RAD51 5UTR R	aagcttGGTAATGCTATTGCTTTCCT	*Hind*III
JM018	RAD51 3UTR F	atgcatGCCTTATGGGTCCGCAACCT	*Nsi*I
JM019	RAD51 3UTR R	ctcgagGCTTCACCGCAGTCATGGCA	*Xho*I
JM020	BRCA2 5UTR F	gcggccgcCGCGCTGAACGGACATGATG	*Not*I
JM021	BRCA2 5UTR R	aagcttGCGGGCCTTAACTGCTGTGA	*Hind*III
JM022	BRCA2 3UTR F	atgcatCGCGTTTGCTGTTGTTGTTG	*Nsi*I
JM023	BRCA2 3UTR R	ctcgagCGGCGTCGATTAACCAACAG	*Xho*I
JM038	Outside RAD51 UTR F	GCATTACTTGATGTGGTGTCGT	
JM039	Outside RAD51 UTR R	CGGAACAATGGAAACCATAACT	
JM041	Outside BRCA2 UTR	CTATGATTGCACGCCGTCGCA	
JM042	Sequence antibiotic	TTGAAGACTTCAATTACACC	
JM044	RAD51 ORF F	GTCGACATGAATACCCGAGCCAAAGG	
JM045	RAD51 ORF R	CCCGGGCTAATCTCTAGCATCTCCGA	
JM046	BRCA2 ORF F	GTCGACATGGTCTTTTCGCAGAAGTC	
JM047	BRCA2 ORF R	GGATCCCTTCCGCATAAGATTGACGA	
JM052	Outside BRCA2 UTR F	GGTCAGCGGGCGGAAGAGGT	
MB40	Hygromycin R	TCTAGATTCCTTTGCCCTCG	

Figure S1

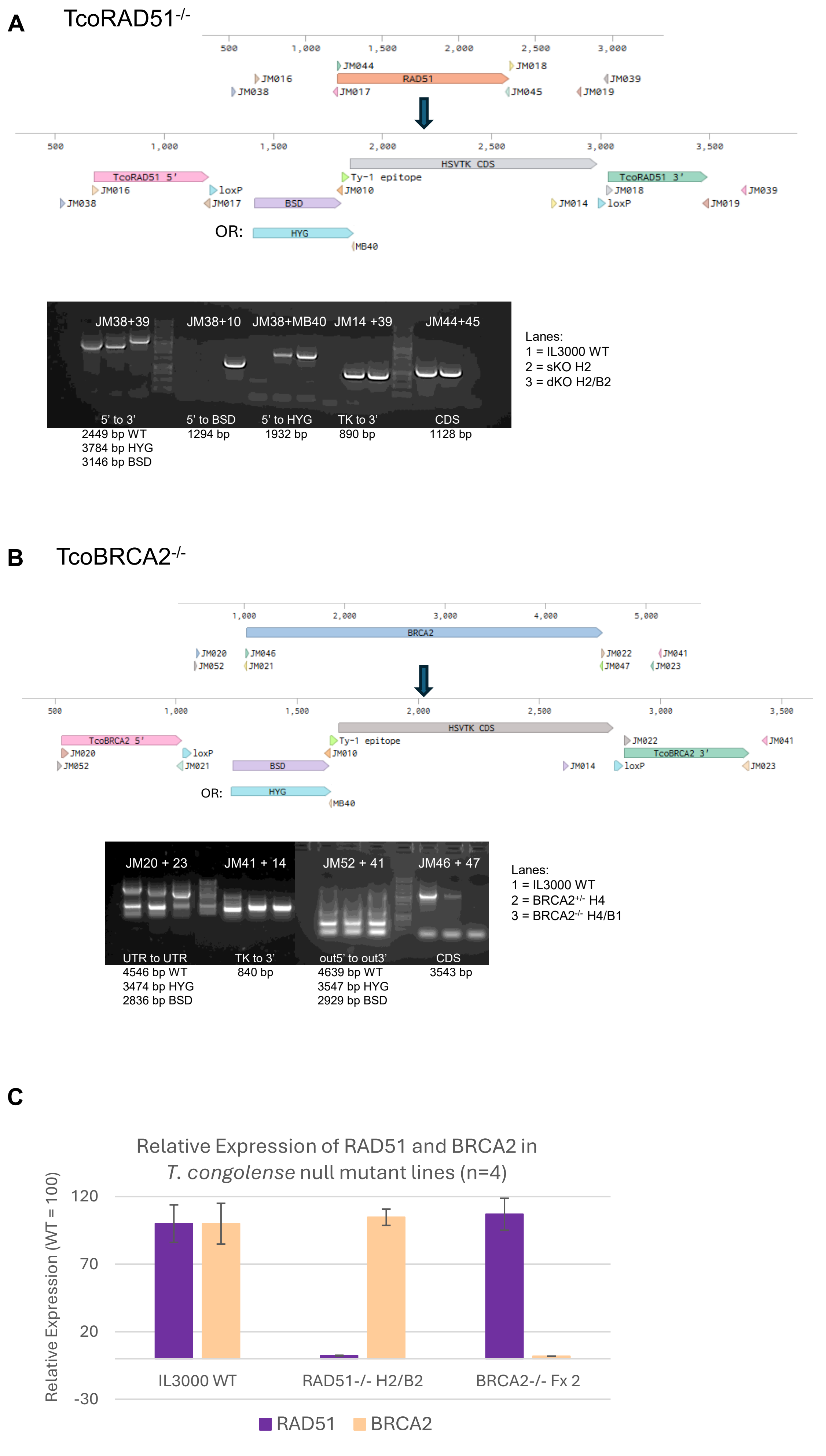
A
TcoRAD51-/-
JM044
JM018
JM016
RAD51
JM039
JM038
JM017
JM045
JM019
TcoRAD51 5'
HSVTK CDS
Ty-1 epitope
TcoRAD51 3'
loxP
JM010
JM014
BSD
OR:
HYG
MB40
JM38+39
JM38+10
JM38+MB40
JM14 +39
JM44+45
5' to 3'
2449 bp WT
3784 bp HYG
3146 bp BSD
5' to BSD
1294 bp
5' to HYG
1932 bp
TK to 3'
890 bp
CDS
1128 bp
Lanes:
1 = IL3000 WT
2 = sKO H2
3 = dKO H2/B2
B
TcoBRCA2-/-
BRCA2
JM020
JM046
JM022
JM041
JM052
JM021
JM047
JM023
TcoBRCA2 5'
TcoBRCA2 3'
JM20 + 23
JM41 + 14
JM52 + 41
JM46 + 47
UTR to UTR
4546 bp WT
3474 bp HYG
2836 bp BSD
TK to 3'
840 bp
out5' to out3'
4639 bp WT
3547 bp HYG
2929 bp BSD
CDS
3543 bp
Lanes:
1 = IL3000 WT
2 = BRCA2+/- H4
3 = BRCA2-/- H4/B1
C
Relative Expression of RAD51 and BRCA2 in
T. congolense null mutant lines (n=4)
Relative Expression (WT = 100)
120
70
20
-30
IL3000 WT
RAD51-/- H2/B2
BRCA2-/- Fx 2
RAD51
BRCA2

Figure S2

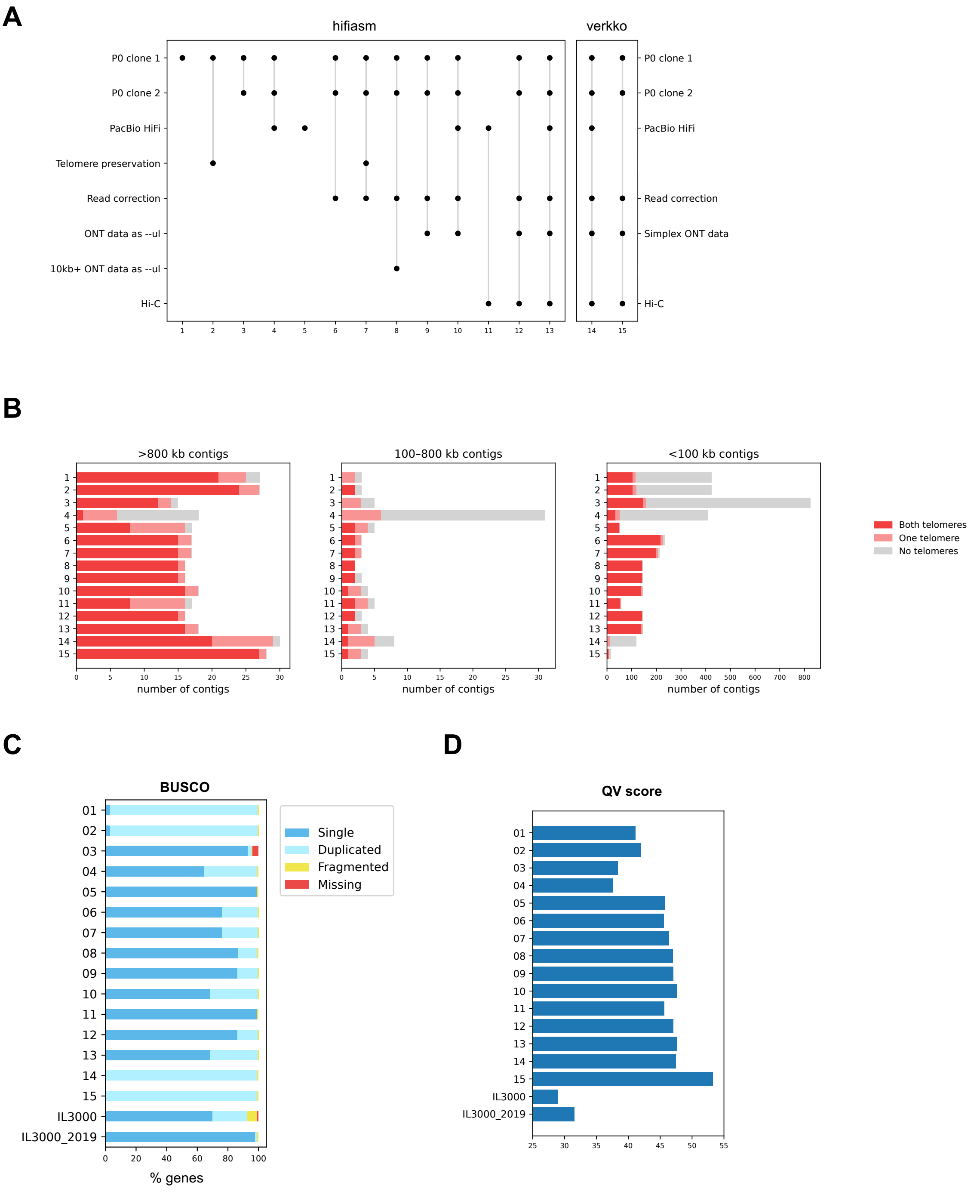
A
hifiasm
verkko
P0 clone 1
P0 clone 2
PacBio HiFi
Telomere preservation
Read correction
ONT data as --ul
10kb+ ONT data as --ul
Hi-C
Simplex ONT data
B
>800 kb contigs
100–800 kb contigs
<100 kb contigs
number of contigs
Both telomeres
One telomere
No telomeres
C
BUSCO
Single
Duplicated
Fragmented
Missing
IL3000
IL3000_2019
% genes
D
QV score

Figure S3

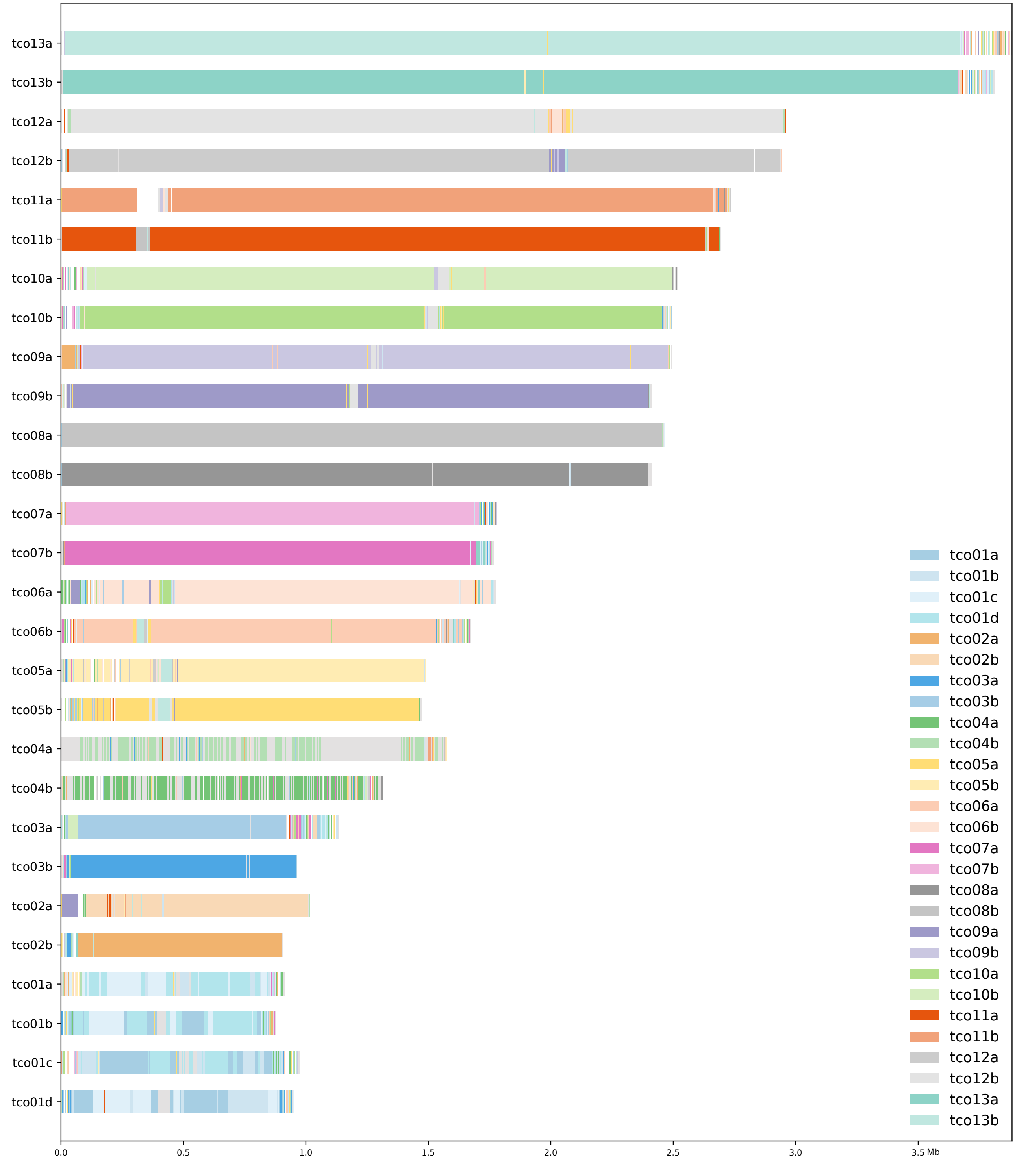
tco13a
tco13b
tco12a
tco12b
tco11a
tco11b
tco10a
tco10b
tco09a
tco09b
tco08a
tco08b
tco07a
tco07b
tco06a
tco06b
tco05a
tco05b
tco04a
tco04b
tco03a
tco03b
tco02a
tco02b
tco01a
tco01b
tco01c
tco01d
0.0
0.5
1.0
1.5
2.0
2.5
3.0
3.5 Mb
tco01a
tco01b
tco01c
tco01d
tco02a
tco02b
tco03a
tco03b
tco04a
tco04b
tco05a
tco05b
tco06a
tco06b
tco07a
tco07b
tco08a
tco08b
tco09a
tco09b
tco10a
tco10b
tco11a
tco11b
tco12a
tco12b
tco13a
tco13b

Figure S4

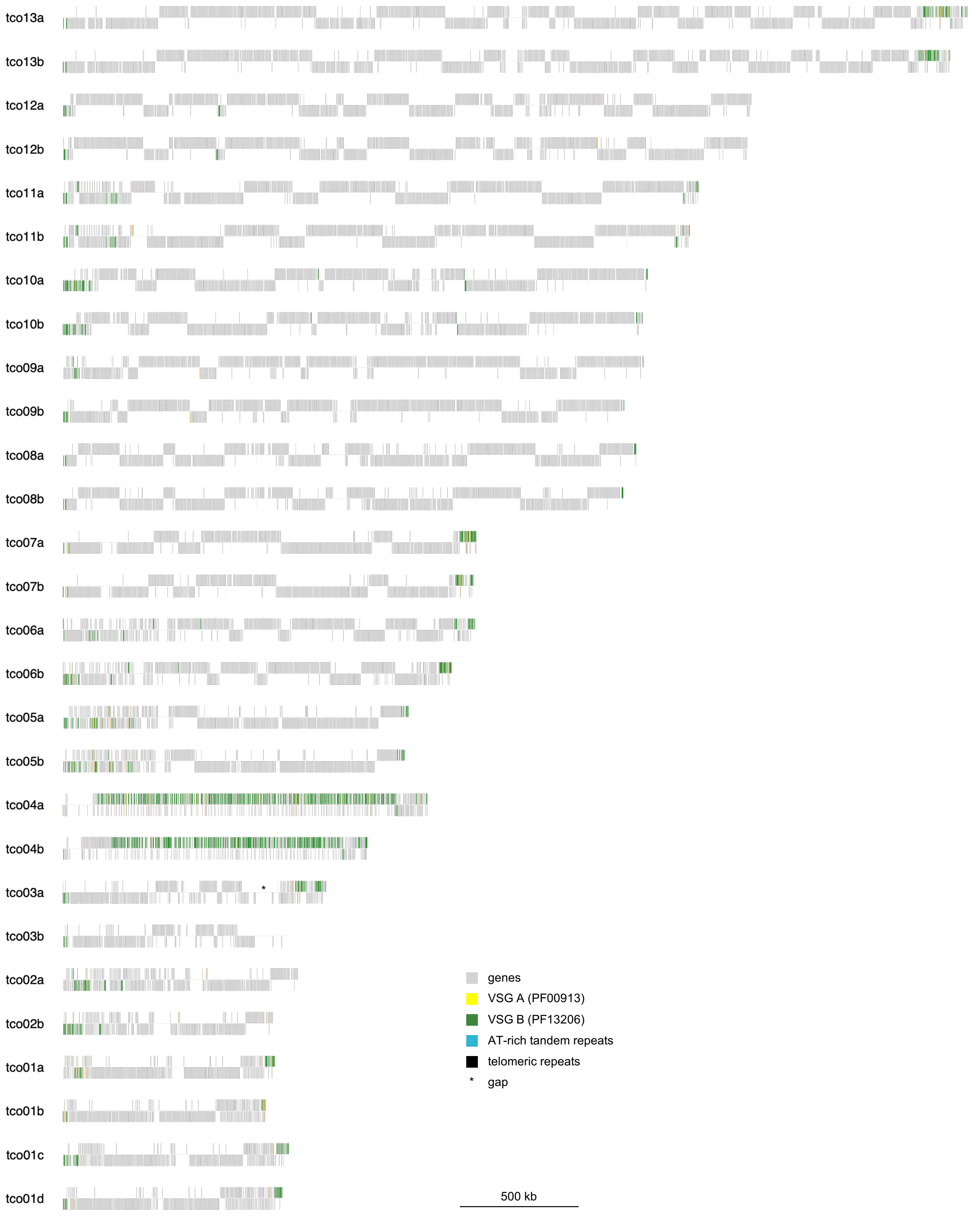

tco13a
tco13b
tco12a
tco12b
tco11a
tco11b
tco10a
tco10b
tco09a
tco09b
tco08a
tco08b
tco07a
tco07b
tco06a
tco06b
tco05a
tco05b
tco04a
tco04b
tco03a
tco03b
tco02a
tco02b
tco01a
tco01b
tco01c
tco01d
*
genes
VSG A (PF00913)
VSG B (PF13206)
AT-rich tandem repeats
telomeric repeats
* gap
500 kb

Figure S5

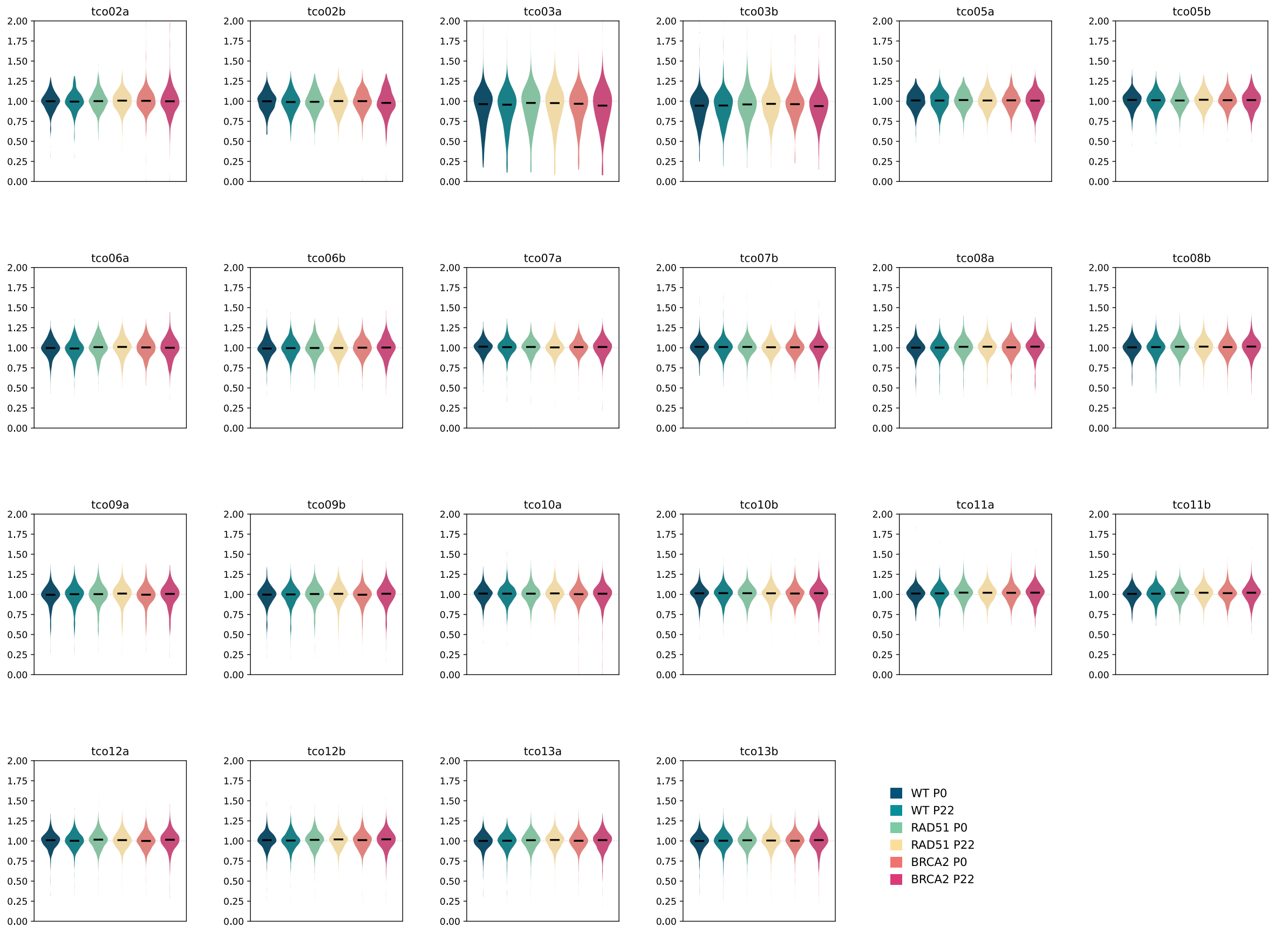
tco02a
tco02b
tco03a
tco03b
tco05a
tco05b
tco06a
tco06b
tco07a
tco07b
tco08a
tco08b
tco09a
tco09b
tco10a
tco10b
tco11a
tco11b
tco12a
tco12b
tco13a
tco13b
0.00
0.25
0.50
0.75
1.00
1.25
1.50
1.75
2.00
WT P0
WT P22
RAD51 P0
RAD51 P22
BRCA2 P0
BRCA2 P22

Figure S6

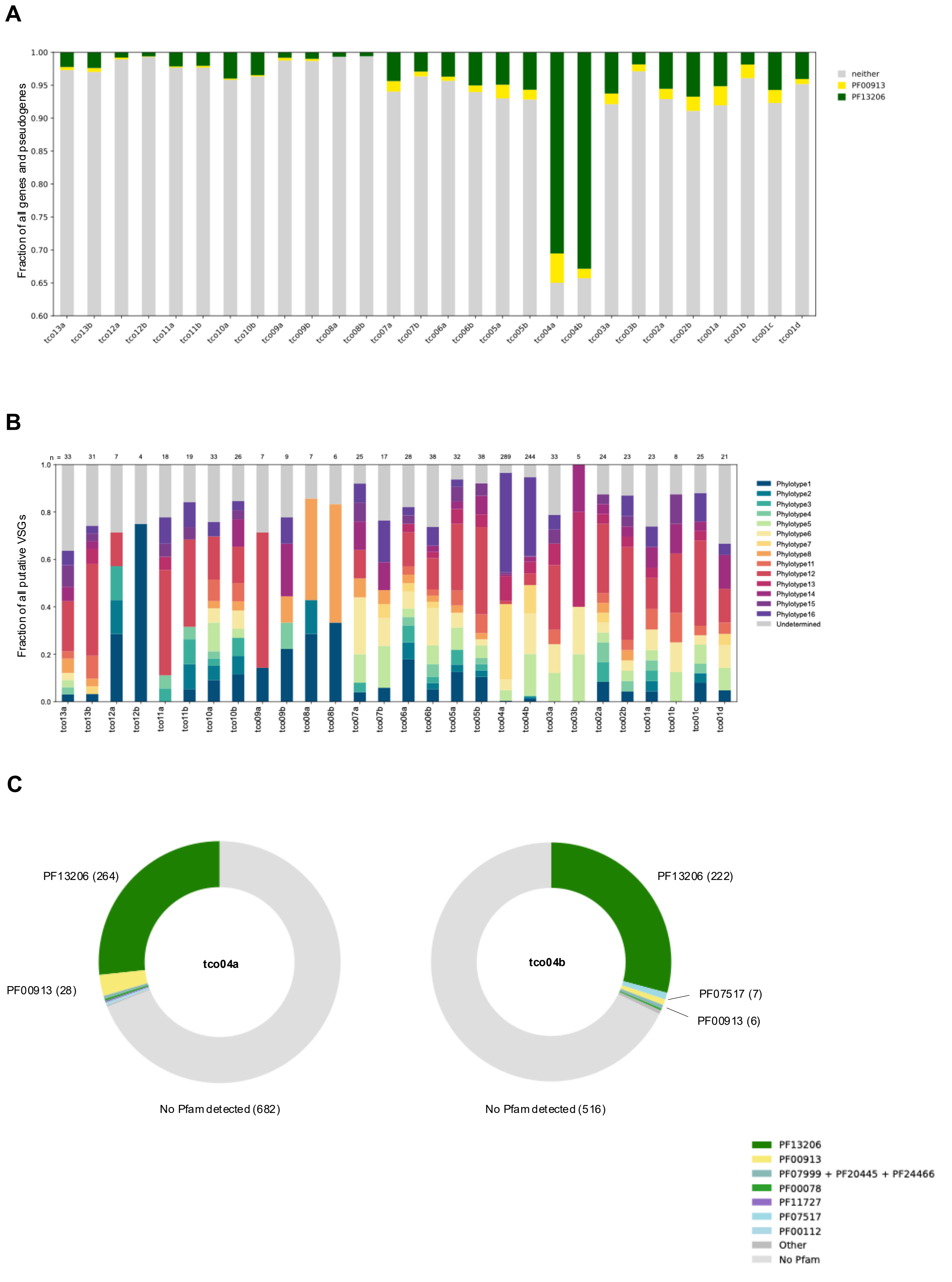
A
Fraction of all genes and pseudogenes
neither
PF00913
PF13206
tco13a tco13b tco12a tco12b tco11a tco11b tco10a tco10b tco09a tco09b tco08a tco08b tco07a tco07b tco06a tco06b tco05a tco05b tco04a tco04b tco03a tco03b tco02a tco02b tco01a tco01b tco01c tco01d
B
n = 33 31 7 4 18 19 33 26 7 9 7 6 25 17 28 38 32 38 289 244 33 5 24 23 23 8 25 21
Fraction of all putative VSGs
Phylotype1
Phylotype2
Phylotype3
Phylotype4
Phylotype5
Phylotype6
Phylotype7
Phylotype8
Phylotype11
Phylotype12
Phylotype13
Phylotype14
Phylotype15
Phylotype16
Undetermined
C
PF13206 (264)
PF00913 (28)
tco04a
No Pfam detected (682)
PF13206 (222)
PF07517 (7)
PF00913 (6)
tco04b
No Pfam detected (516)
PF13206
PF00913
PF07999 + PF20445 + PF24466
PF00078
PF11727
PF07517
PF00112
Other
No Pfam

Figure S7

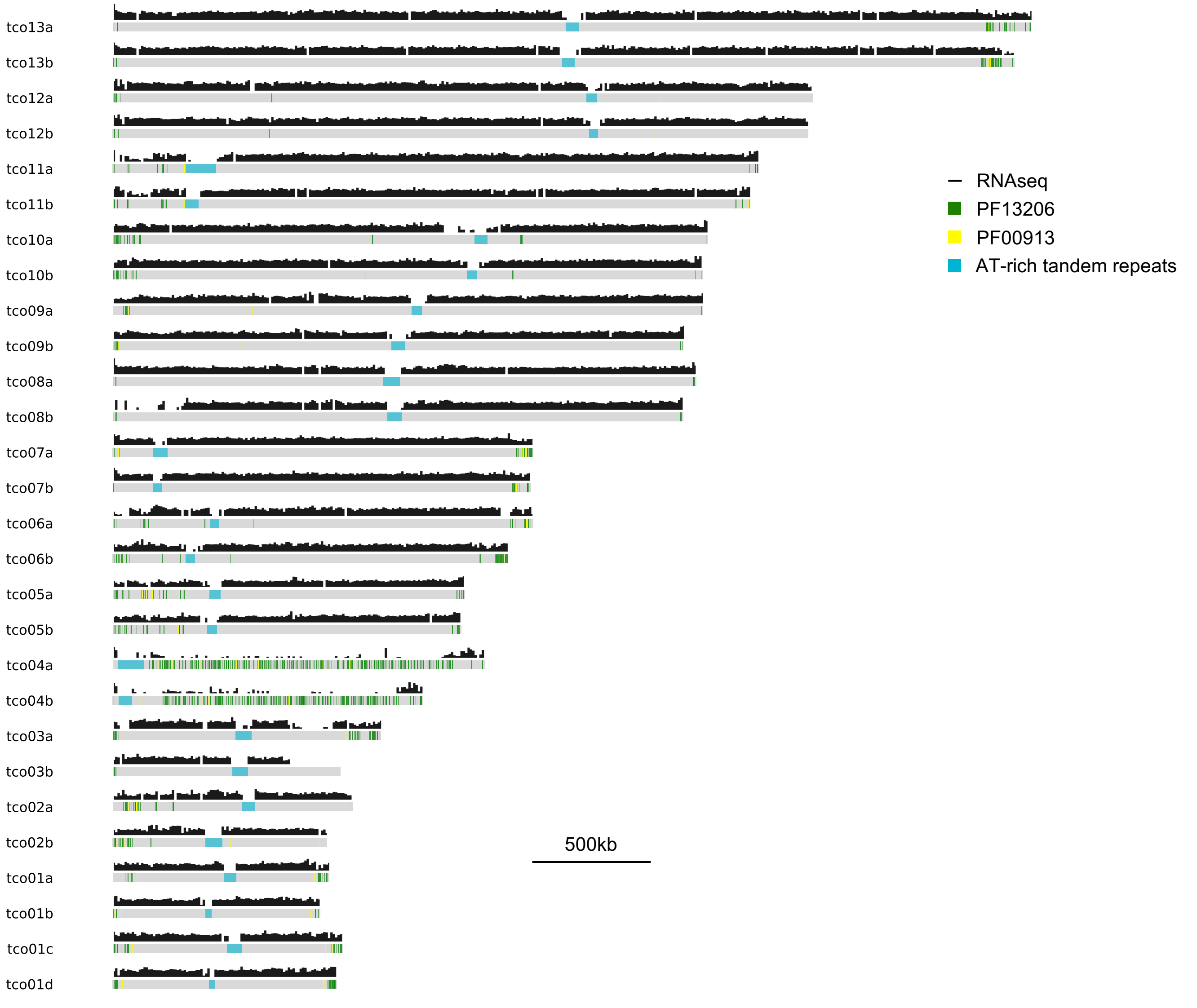
tco13a
tco13b
tco12a
tco12b
tco11a
tco11b
tco10a
tco10b
tco09a
tco09b
tco08a
tco08b
tco07a
tco07b
tco06a
tco06b
tco05a
tco05b
tco04a
tco04b
tco03a
tco03b
tco02a
tco02b
tco01a
tco01b
tco01c
tco01d
RNAseq
PF13206
PF00913
AT-rich tandem repeats
500kb

Figure S8

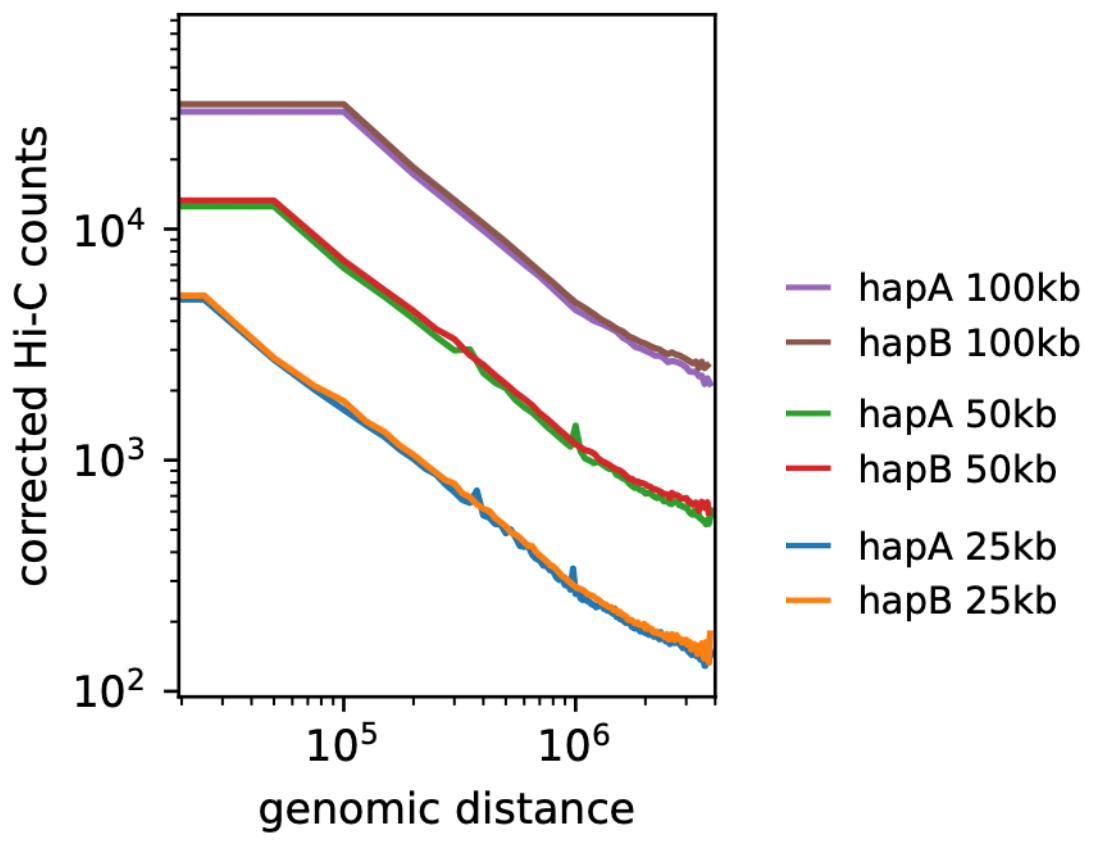
corrected Hi-C counts
10^4
10^3
10^2
10^5
10^6
genomic distance
hapA 100kb
hapB 100kb
hapA 50kb
hapB 50kb
hapA 25kb
hapB 25kb

Figure S9

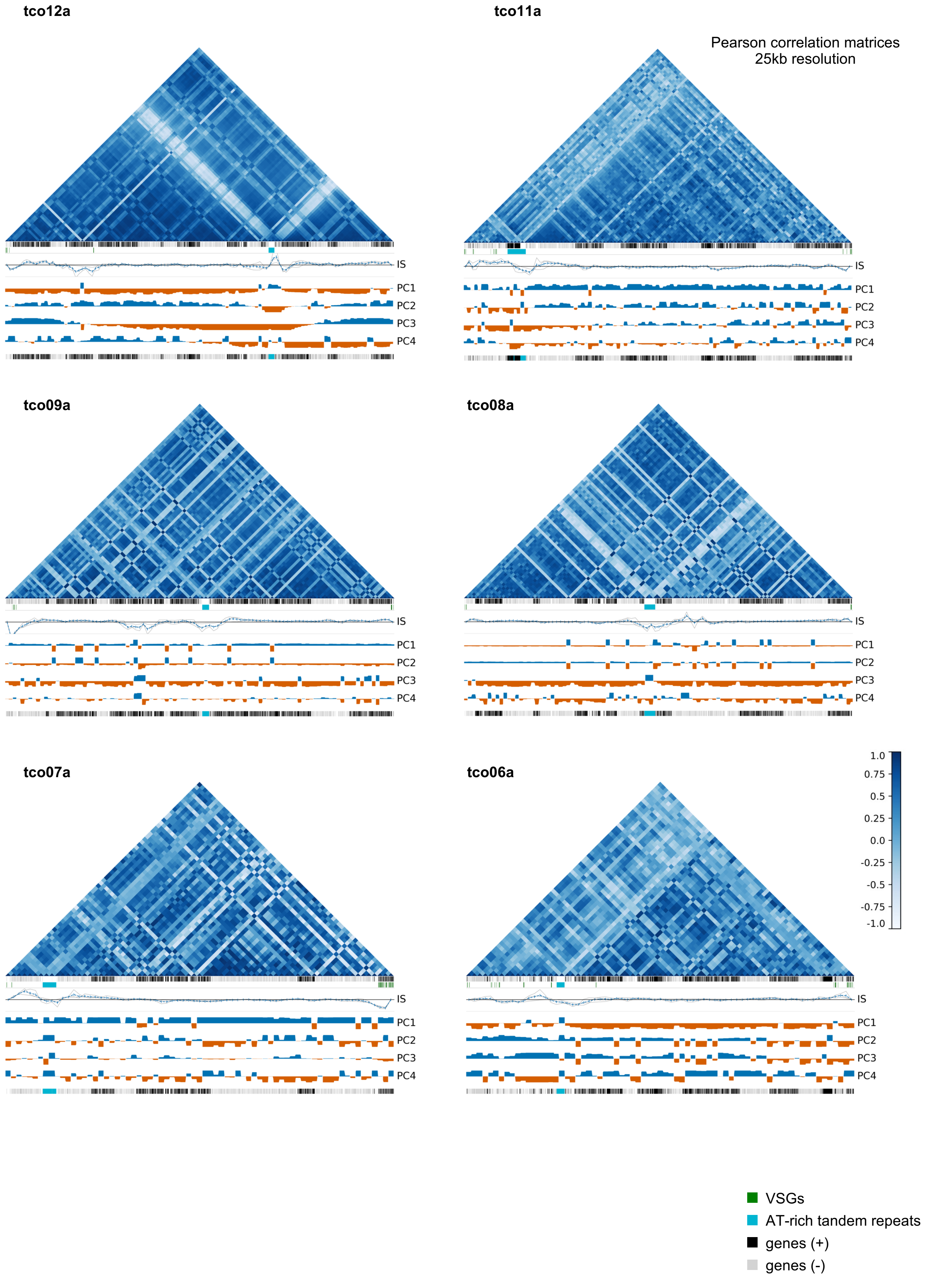

tco12a
tco11a
Pearson correlation matrices
25kb resolution
tco09a
tco08a
tco07a
tco06a
IS
PC1
PC2
PC3
PC4
1.0
0.75
0.5
0.25
0.0
-0.25
-0.5
-0.75
-1.0
VSGs
AT-rich tandem repeats
genes (+)
genes (-)

Figure S9 continued

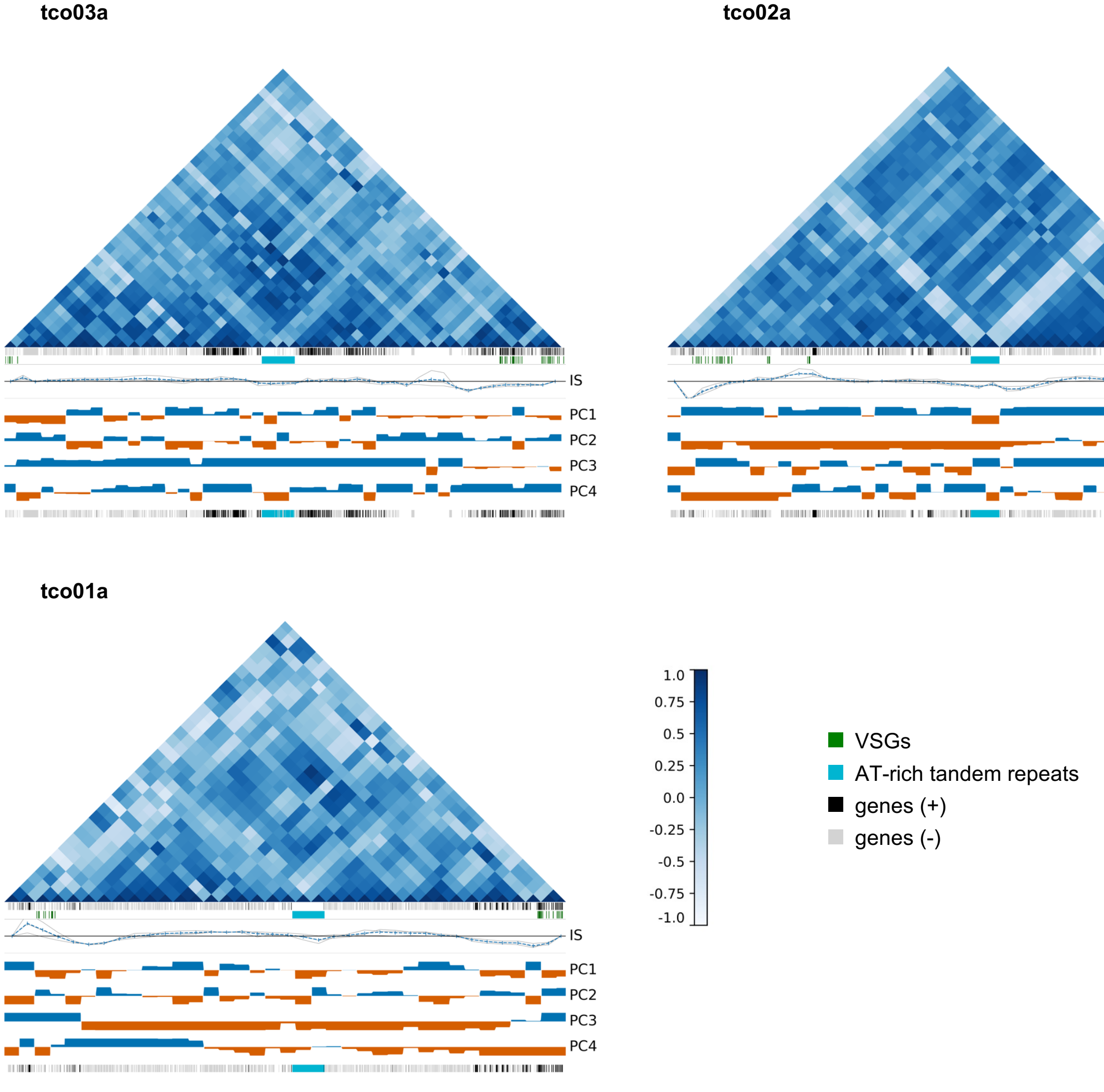

Figure S10

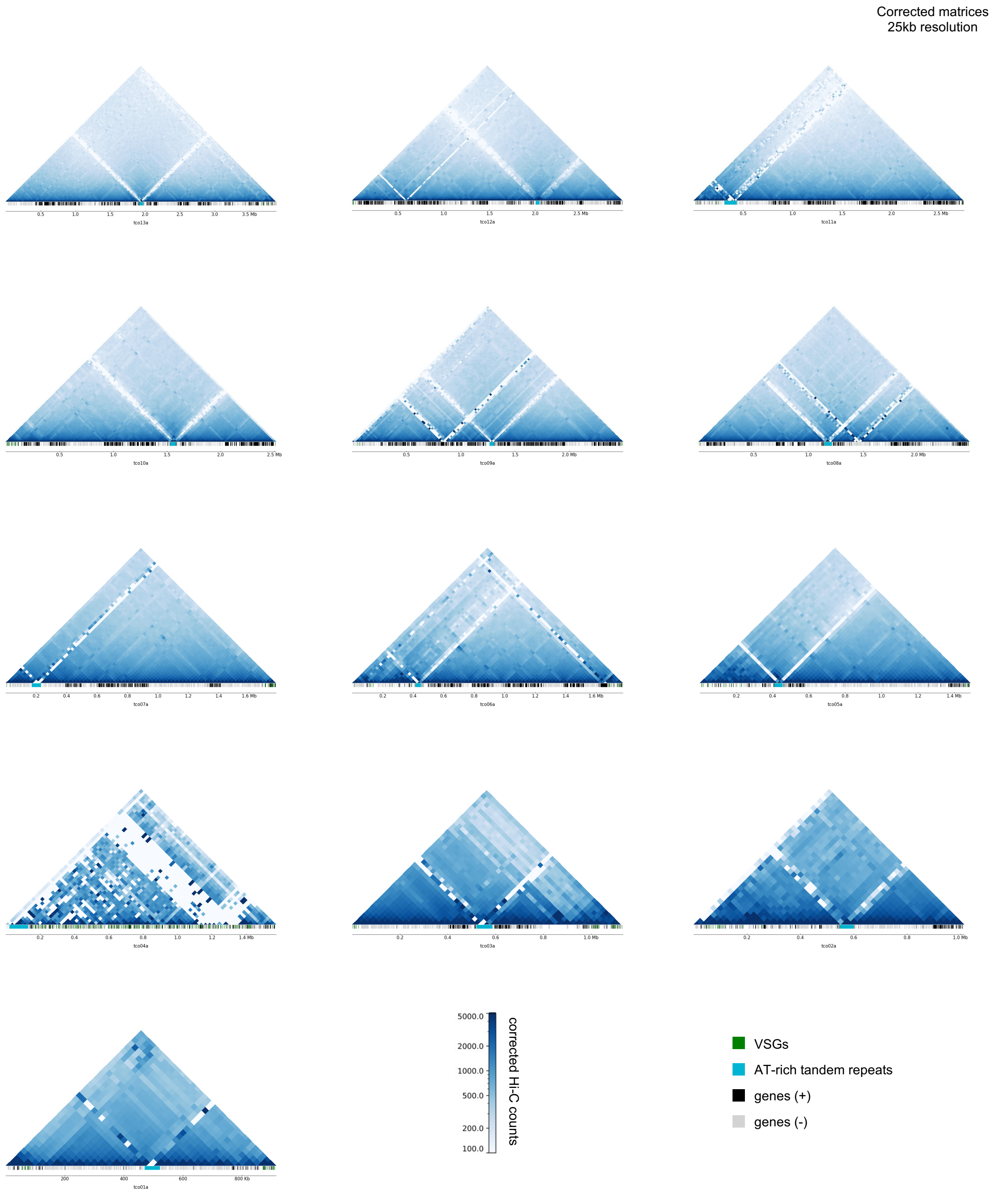
Corrected matrices
25kb resolution
tco13a
tco12a
tco11a
tco10a
tco09a
tco08a
tco07a
tco06a
tco05a
tco04a
tco03a
tco02a
tco01a
5000.0
2000.0
1000.0
500.0
200.0
100.0
corrected Hi-C counts
VSGs
AT-rich tandem repeats
genes (+)
genes (-)

Table S1

	IL3000	IL3000_2019	2026
# contigs (>= 0 bp)	2839	375	189
# contigs (>= 1000 bp)	2834	375	189
# contigs (>= 5000 bp)	1289	359	189
# contigs (>= 10000 bp)	503	312	189
# contigs (>= 25000 bp)	77	185	188
# contigs (>= 50000 bp)	16	113	44
Total length (>= 0 bp)	41372041	39170526	61650343
Total length (>= 1000 bp)	41368191	39170526	61650343
Total length (>= 5000 bp)	36540844	39113032	61650343
Total length (>= 10000 bp)	30997589	38767969	61650343
Total length (>= 25000 bp)	24679909	36629074	61636704
Total length (>= 50000 bp)	22805545	34025332	56387238
# contigs	2839	375	189
Largest contig	4815855	4601516	3883400
Total length	41372041	39170526	61650343
GC (%)	48.18	45.98	47.45
N50	1222280	1070342	2412744
N90	4531	39286	192563
auN	1512764.1	1614293.1	2064127.1
L50	9	9	11
L90	1435	141	32
# N's per 100 kbp	17624.70	189.21	1.62
# predicted genes (unique)	15046	15879	20812
# predicted genes (>= 0 bp)	15869 + 347 part	16433 + 16 part	24000 + 1 part
# predicted genes (>= 300 bp)	13532 + 329 part	14091 + 15 part	21299 + 1 part
# predicted genes (>= 1500 bp)	4124 + 117 part	3946 + 3 part	7435 + 0 part
# predicted genes (>= 3000 bp)	1060 + 37 part	999 + 1 part	2239 + 0 part
# predicted rRNA genes	73 + 9 part	134 + 22 part	256 + 32 part

Table S2

Assembly completeness metrics

	hifiasm	verkko	combined	tools, versions, settings
**k-mer completeness**	85.9275	92.9124	96.9925	merqury, v.1.4.1
**QV**	47.0157	53.2681	47.1202	merqury, v.1.4.1
**BUSCO (%)**				busco, v. 5.8.3, euglenozoa_odb12 (3611 markers)
**complete**	93.9	94.4	94.2	
**single**	80.1	1.5	1	
**duplicated**	13.8	93	93.2	
**fragmented**	2.2	2	2.1	
**missing**	3.8	3.5	3.7	
**internal stop**	1	0.6	0.6	

Mapped read percentage to combined assembly

**Short paired-end reads (BGI)**	95.06
**Long read simplex data (ONT)**	87.58
**Long read HiFi data (PacBio)**	99.99

Assembly split by chromosome or contig type

Megabase	Intermediate	Mini	Maxicircle	Unassigned
54.41 Mb	650 kb	5.46 Mb	28 kb	1.1 Mb
88.25%	1.06%	8.86%	0.05%	1.79%
